# Investigation of Biological Activity of Fucoidan and Laminarin as Bioactive Polysaccharides from Irish Brown Macroalgae

**DOI:** 10.3390/cells13231938

**Published:** 2024-11-22

**Authors:** Shanmugapriya Karuppusamy, Janith Wanigasekara, Stephen Fitzpatrick, Henry Lyons, James Curtin, Gaurav Rajauria, Brijesh K. Tiwari, Colm O’Donnell

**Affiliations:** 1 School of Biosystems and Food Engineering, University College Dublin, D04 V1W8 Dublin, Ireland; colm.odonnell@ucd.ie; 2 School of Food Science and Environmental Health, College of Sciences and Health, Technological University Dublin, D01 K822 Dublin, Ireland; d18128066@mytudublin.ie (J.W.); james.curtin@tudublin.ie (J.C.); 3 Nutramara Ltd., Beechgrove House Strand Street, V92 FH0K Tralee, Ireland; s.fitzpatrick@nutramara.com (S.F.); h.lyons@nutramara.com (H.L.); 4 School of Microbiology, School of Food and Nutritional Sciences, SUSFERM Centre for Fermentation and Bioprocess Engineering, University College Cork, T12 K8AF Cork, Ireland; grajauria@ucc.ie; 5 Department of Food Chemistry and Technology, Teagasc Food Research Centre, D15 KN3K Dublin, Ireland; brijesh.tiwari@teagasc.ie

**Keywords:** seaweed, laminarin, fucoidan, antioxidant, anti-inflammatory, antidiabetic, cytotoxicity, 3D tumour spheres

## Abstract

This study aimed to investigate the biological activity of crude and purified laminarin and fucoidan samples extracted from Irish brown macroalgae species *Laminaria digitata* and *Fucus vesiculosus*. The antioxidant capacity of the samples was evaluated using the 2,2-diphenyl-1-picrylhydrazyl and ferric-reducing antioxidant power assays. The anti-inflammatory potential of the samples was analysed using the cyclooxygenases inhibition activity, and the antidiabetic activity was evaluated using a dipeptidyl peptidase-4 inhibitor screening assay. The cytotoxicity of the samples was measured using the Alamar Blue™ assay with different types of cancer cell lines. The crude laminarin and fucoidan samples exhibited higher antioxidant activity (*p* < 0.05) than the purified samples and commercial standards. Similarly, the crude extracts showed stronger anti-inflammatory and antidiabetic effects compared to the purified samples. Additionally, the crude laminarin and fucoidan samples showed higher cytotoxic activity. Specifically, as confirmed in the flow cytometry analysis, 3D tumour spheres using different cancer cell lines showed significantly higher resistance to bioactive compounds compared to 2D monolayer cells. The laminarin and fucoidan polysaccharide samples investigated are suitable for potential nutraceutical applications based on the biological activity values observed. Future research is necessary to purify the bioactive compounds investigated and improve their selectivity for targeted therapeutic uses in food and biomedical applications.

## 1. Introduction

Cancer is a leading cause of mortality worldwide, with nearly 10 million deaths reported in 2020. According to the World Health Organization (WHO), the types of cancer with the greatest prevalence are lung, liver, stomach, colorectal, mammary, and oesophageal cancer [1,2,3,4]. Potential cancer treatments include surgery, radiotherapy, and/or systemic therapy (chemotherapy, hormonal treatments, and targeted biological therapies). Glioblastoma (GBM) is the most common and aggressive malignant primary brain tumour reported in adults. The WHO 2021 classification reports that the term GBM includes isocitrate dehydrogenase (IDH) wild-type tumours [4]. Reported treatments for GBM include surgical resection, radiation, and chemotherapy; however, the outcomes are poor due to high invasiveness, high resistance to chemotherapy, and the inability to traverse the blood–brain barrier, which leads to a low survival rate in human patients [5].

Cancer is characterised by increased oxidative stress and an imbalance between reactive oxygen species (ROS) and antioxidants [6]. ROS in brown seaweeds have been characterised to act as secondary messengers involved in regulating metabolic processes at the cellular level [7]. ROS react with biological molecules to contribute to processes related to cellular signalling that lead to oxidative stress. The main cellular components are affected by various types of oxidative stress, leading to the development of various diseases/pathologies, such as arteriosclerosis, cardiovascular and neurodegenerative diseases, diabetes, rheumatism, and cancer [8,9]. In the later stages, ROS in cancer cells can be attributed to the strong genetic or mutagenic effects in terms of oxidative DNA damage and genomic instability [6]. This novel therapeutic approach becomes an attractive concept in the use of antioxidants for the treatment of cancer, in which oxidative stress plays a significant role in carcinogenesis and cancer progression [10]. Multiple antioxidant therapeutic strategies, such as those using vitamin E, ubiquinol, glutathione, and superoxide dismutase, have been explored, with great interest in developing numerous enzymatic and non-enzymatic antioxidants as anticancer drugs in pre-clinical and clinical research [11].

Different natural dietary antioxidants have been explored to prevent the deleterious effects produced by oxidative stress, which favour the use of seaweed bioactives or polysaccharides as one of them [10]. In this respect, brown macroalgae are well known to be a reliable source of natural antioxidant compounds and possess efficient defence systems that can tolerate a wide array of oxidative stress-inducing factors [12]. Therefore, marine macroalgae have been extensively studied for their potential biological and therapeutic effects, due to their strong ability to synthesise primary and secondary metabolites (polyphenolic compounds, sterols, vitamins, polysaccharides, and polyunsaturated fatty acids) of biological and biomedical importance [13]. Many studies have reported the biological properties of the brown seaweed polysaccharides laminarin and fucoidan [12,14,15,16]. The free radicals include reactive oxygen species (ROS) and reactive nitrogen species (RNS). Reactive oxygen species include superoxide anion (O_2_ ^·−^) and nitric oxide (NO) radicals. Non-radical derivatives of oxygen include hydrogen peroxide (H_2_O_2_) and singlet oxygen (^1^O_2_) [17,18,19].

Seaweed polysaccharides also possess antidiabetic, anti-inflammatory, and anticoagulant activity [20,21]. Previous studies have reported the physical, chemical, and functional characteristics of crude seaweed extracts generated by using green technology and nanotechnological aspects for drug delivery and other pharmaceutical-related applications [19,22]. In addition, the chemical compounds present in seaweed were also found to induce various biological effects, such as oxidative damage/breakage to DNA, structural damage to cells, the generation of different cell signalling pathways, allergic reactions, respiratory disorders, direct and indirect protein oxidation by ROS and oxidative stress, induced apoptosis, and cancer prevention [22,23,24]. Therefore, various physical, chemical, and functional characteristics are being explored. Numerous gaps have been identified regarding the biological potential of seaweed in preventing diseases. Both non-steroidal anti-inflammatory drugs (NSAID) and steroidal anti-inflammatory drugs (SAID) are extensively used for the treatment of the majority of diseases accompanied by acute and chronic inflammation, but these types of drugs are well known to have some adverse side effects. Seaweed polysaccharides have great potential for the discovery of new natural drugs with anti-inflammatory activity but with relatively low incidences of side effects [19,25]. Concerning the high interest in the inflammatory mediator process, seaweed polysaccharides have specific potential to affect the course of both acute and chronic inflammation processes.

In recent decades, uncontrolled glycaemic levels in patients with diabetes have become one of the main causes of death in humans [26]. Specifically, dipeptidyl peptidase-4 (DPP-4) is an enzyme that is involved in the inhibition of the rapid degradation of hormones, which prevents hyperglycaemia [27,28]. In addition, it reduces glucose production and increases insulin production. Therefore, DPP-4 inhibitors have played a vital role in the diabetic field as a new therapy. The antidiabetic activity of fucoidan and laminarin has been reported in the literature [29,30,31]; however, their mechanisms have not been proven. Owing to their chemical structures, seaweed polysaccharides are considered complex macromolecules with broad action mechanisms [14,16]. Therefore, the various biological properties of seaweed polysaccharides are the focus of the present study.

The objective of this study was to investigate the biological activity of crude and purified laminarin and fucoidan as seaweed polysaccharides and evaluate their cytotoxicity in different cancer cell lines.

## 2. Materials and Methods

### 2.1. Chemicals

Hydrochloric acid, 2,2-diphenyl-1-picrylhydrazyl (DPPH), 2,4,6-tripyridyl-s-triazine (TPTZ), and 6-hydroxy-2,5,7,8-tetramethylchroman-2-carboxylic acid (Trolox) were purchased from Sigma (Sigma-Aldrich, Saint Louis, MO, USA). Ultrapure water was used in all extraction procedures and chemical analyses. All chemicals used for the anticancer study were supplied by Sigma-Aldrich, unless stated otherwise.

### 2.2. Extraction of Seaweed Samples

The crude laminarin and fucoidan samples from *Laminaria digitata* and *Fucus vesiculosus* species were obtained from Nutramara Ltd. (Tralee, Co., Kerry, Ireland). Laminarin and fucoidan standards were purchased from Sigma-Aldrich (Merck Life Science Limited, Co., Wicklow, Ireland). The crude samples were extracted using green novel extraction processes such as ultrasound extraction and characterised in previous studies [12,15]. The molecular weight cut-off (MWCO) fraction for the crude seaweed extract was prepared according to a multi-step purification process for selected dried brown seaweeds [32]. The seaweed samples used for this study were crude laminarin (C.L.), an MWCO 10 kDa laminarin fraction (M.L.), a Sigma laminarin standard (S.L.), crude fucoidan (C.F.), an MWCO 10 kDa fucoidan fraction (M.F.), and a Sigma fucoidan standard (S.F.). Freeze-dried crude extracts and MWCO purified fractions were used in *in vitro* biological studies, including studies of their antioxidant, antidiabetic, anti-inflammatory, wound-healing, and anticancer properties.

### 2.3. Antioxidant Activity

The antioxidant properties of the extracts were measured using the 2,2-diphenyl-1-picrylhydrazyl radical scavenging activity (DPPH assay) and ferric-reducing antioxidant power (FRAP assay). The most common assays to investigate seaweed bioactives are the DPPH and FRAP assays, both according to a test reaction after an electron-deficient radical. The DPPH assay is based on the reduction of the purple DPPH· to 1,1-diphenyl-2-picryl hydrazine, and the FRAP assay deals with those containing ferrous ions in a known concentration. All experiments were carried out in triplicate, with three readings of each replicate (*n* = 9).

#### 2.3.1. DPPH Radical Scavenging Assay

The DPPH assay was performed according to the method of Garcia-Vaquero et al. [33] and modified by Shanmugapriya et al. [9]. Briefly, seaweed extracts and ascorbic acid as a positive control were prepared in 0.1 M citrate phosphate buffer with 0.3% of Triton X-100. The samples were prepared in a triplicate manner. Then, 10 μL of a 2 mM methanolic DPPH solution was added to each well. Further, the reaction mixture was incubated at room temperature in the dark for 30 min and measured at 515 nm before and after the reaction with the DPPH solution using a UV–Vis spectrophotometer. The inhibition percentage of DPPH scavenging activity was calculated using Equation (1).

(1) $$DPPH\;radical\;scavenging\;activity\;\left(\%\right)=\frac{{Abs}_{Blank}-{Abs}_{Sample}}{{Abs}_{Blank}}⨯100\;$$

where “Abs*_Blank_*” is the absorbance of the blank (DPPH solution without sample/standard), and “Abs*_Sample_*” is the absorbance of the test sample (DPPH solution plus test sample/standard). The IC_50_ values corresponding to the percentage of radical scavenging capacity (% RSC) represent the concentration of the extract that causes 50% neutralisation, which was calculated by linear regression analysis. All measurements were performed in triplicate.

#### 2.3.2. Ferric-Reducing Antioxidant Power (FRAP) Assay

The FRAP assay was performed following the method proposed by Dudonné et al. [34] and Henderson et al. [35]. Briefly, seaweed extracts and Trolox as a standard at concentrations ranging from 15 to 420 μM were prepared. The FRAP solution was freshly prepared with 300 mM acetate buffer (pH 3.6), 20 mM ferric chloride, 10 mM 2,4,6-Tripyridyl-s-Triazine (TPTZ) in 40 mM HCl, and Milli Q water. Then, 280 μL of a FRAP working solution was added to 20 μL of each sample and they were incubated at 37 °C in the dark for 30 min; then, the absorbance was read at 593 nm using a UV–Vis spectrophotometer. The FRAP antioxidant activity values were expressed as µM Trolox equivalent antioxidant capacity (TE) per mg of extract.

### 2.4. Immune Stimulation and Inflammatory Activity

The screening of the anti-inflammatory activity of the samples against cyclooxygenase (COX)-1 and -2 was performed using an enzyme inhibition screening assay kit for COX-1/2, with item no. 560131, purchased from Cayman Chemical (Cayman Chemical Company, Ann Arbor, MI, USA). The procedure outlined in the kit was followed, with slight modifications, in triplicate (n = 9) [36,37]. Diclofenac sodium (1 mg/mL) was used as a reference compound in the assay. Cyclooxygenase-1 (COX-1) and COX-2 are bifunctional enzymes; they were used for the anti-inflammatory analysis of the samples. COX-1 is responsible for undesirable gastrointestinal and renal side effects. COX-2 is responsible for the biosynthesis of PGs under acute inflammatory conditions. The COX formulations were prepared as follows: 80 μL of ovine COX-1 and human recombinant COX-2 enzymes was diluted with 320 μL of diluted reaction buffer and stored at −80 °C. In the COX dilutions, two test tubes, labelled BC1 and BC2, were taken to prepare background samples. The samples for 100% initial activity were diluted and labelled IA1, IA2, and IA3. Similarly to the 100% initial activity dilutions, the inhibitor samples were diluted and labelled C1–C3. Specifically, the sample background COX-1/COX-2 tubes/wells and 100% initial activity tubes/wells were prepared for COX reactions. All tubes were incubated for 10 min at 37 °C, and then 10 µL arachidonic acid was added to all of the reaction tubes. After incubation, a saturated stannous chloride solution was added for enzyme catalysis and the samples were maintained at 4 °C. For the enzyme immunoassay procedure, the buffers and assay-specific reagents were prepared. The reaction dilutions were then placed in a 96-well plate for the assay, as per the protocol of the kit. The plate was covered with plastic film and incubated for 18 h at RT, and we followed the procedure for the development of the plate by washing each well with a wash buffer and Ellman’s reagent. The tracer was added to the total activity (TA) well. Then, the plate was incubated for 1 h in the dark. The absorbance was measured at 420 nm. The % B/Bo (% sample or maximum bound) values for the standards and each sample were calculated from the absorbance values obtained. The number of prostaglandins (PG) formed was calculated from the values of % B/Bo and the standard curve. The percentage inhibition values of the standards and samples were calculated using the inhibitor concentration to determine the IC_50_ values. The IC_50_ value is defined as the concentration of an inhibitor where the response is reduced to 50% (half the maximal inhibitory concentration) of the desired compound. The IC_50_ values and selectivity index (SI) values for the compounds were analysed using the percent inhibition values to determine the inhibitory effects of the seaweed samples.

### 2.5. In Vitro Gastrointestinal Digestion

The in vitro gastrointestinal digestion model was implemented in the seaweed samples, as described by Khalid et al. [38]. This assay was performed with a DPP-IV inhibitor screening kit (ab133081, Cambridge, UK, Abcam). The cleavage of the peptide bond by dipeptidyl peptidase (DPP) releases the free Gly-Pro-Aminomethylcoumarin (AMC) group, resulting in fluorescence, which can be analysed using an excitation wavelength of 350–360 nm and an emission wavelength of 450–465 nm. The inhibitory activity of the samples against DPP-IV in the presence of Gly-Pro-AMC with different concentrations was carried out and presented in terms of the IC_50_. The seaweed samples were dissolved in the assay buffer provided and diluted to concentrations ranging from 100 to 1000 µg/mL. The diluted samples were added to the wells of 96-well plates with opaque walls and transparent bottoms. In addition, the DPP-IV enzyme was diluted with an assay buffer in the ratio of 1:4 (*v*/*v*). The initial activity wells contained 40 μL of assay buffer, 10 μL diluted DPP-IV, and 50 μL substrate. Sitagliptin, as a positive control, was prepared as stock solutions of 10% *w*/*v*. Then, the inhibitory activity wells were prepared with 10 μL of the sample/control, 30 μL of assay buffer, 10 μL of DPP-IV, and 50 μL of the substrate. The reaction was initiated by the addition of 20 μL of bovine serum solution, and the samples were incubated at 37 °C with agitation for 1 h. Then, the plate was covered with a 96-well cover sheet and sealed with aluminium foil; then, it was again incubated for 30 min at 37 °C. After incubation, the fluorescence readings were recorded using a multi-mode microplate reader at an excitation wavelength of 355 nm and an emission wavelength of 460 nm. The average fluorescence of the 100% initial activity, background, and inhibitor wells was determined. The fluorescence of the background wells was subtracted from the fluorescence of the 100% initial activity and inhibitor wells. The inhibitory activity was calculated using Equation (2).

(2) $$Percentage\;inhibition=\frac{\left(Initial\;activity-Inhibitory\;activity\right)}{\left(Initial\;activity\right)}\times 100\;$$

### 2.6. Anticancer Activity

#### 2.6.1. Cell Culture

The U-251MG human glioblastoma cell line (formerly known as U-373 MG-CD14) was obtained from Trinity College Dublin (Dublin, Ireland). Other cell lines, namely the A431 human epidermoid carcinoma, HepG2 human hepatoma, Caco-2 human colorectal adenocarcinoma, and HEK293 human embryonic kidney cell lines, were purchased from the ATCC European Distributor (LGC Standards, Dublin, Ireland). The mentioned cell lines were selected based on the biofunctional potential of seaweed bioactives for human health. The mycoplasma was tested using a MycoAlert^®^ PLUS Mycoplasma Detection Kit (Lonza Bioscience, Cologne, Germany).

#### 2.6.2. Two-Dimensional Cell Culture Method

All cell cultures mentioned above were used for the 2D cell culture method. Cells were cultured in Dulbecco’s Modified Eagle’s Medium high glucose (DMEM) supplemented with 10% foetal bovine serum (FBS) and 1% penicillin–streptomycin as antibiotics and maintained in a humidified incubator containing 5% CO_2_ at 37 °C in a T75 standard flask (Sarstedt AG & Co. KG, 51588 Nümbrecht, Germany). Cells were subcultured with 0.25% *w*/*v* trypsin–EDTA solution until 70–80% confluence was reached. Cells were seeded at the desired density (1–2.5 × 10^3^ cells/well) for 2 days, followed by 6 days of treatment in triplicate, and were incubated overnight at 37 °C with 5% CO_2_. The cells were treated with 1% of 10% *w*/*v* (in µg/mL) as a stock concentration for each of the different laminarin and fucoidan fractions and serially diluted from 1% to 0.0078%, whereas 20% dimethyl sulfoxide (DMSO) was used as a positive control and media were used as a negative control.

#### 2.6.3. Three-Dimensional Cell Culture Method

Human glioblastoma cell (U-251MG) suspensions with the desired seeding density were seeded into Nunclon™ Sphera™ 96-well low-attachment plates (Thermo Fisher Scientific, Waltham, Massachusetts 02451, USA) in DMEM high glucose supplemented with 10% FBS and 1% penicillin–streptomycin. The low-attachment plates were centrifuged at 1250 rpm for 5 min and incubated at 37 °C with 5% CO_2_ and 95% humidity, as described in Wanigasekara et al. [39,40]. Tumour spheroid formation was observed within 4 days and confirmed at regular intervals using an Optika XDS-2 trinocular inverse microscope equipped with an ISH500 camera. Fresh media were added every three days by replenishing the old media in each well, without disturbing the tumour spheroids. After tumour spheroid formation, the seaweed extracts were treated and incubated for 144 h. The tumour spheres were treated with each of the different laminarin fractions and serially diluted from 1% to 0.0078%, whereas 20% DMSO was used as positive control and DMEM media with FBS were used as a negative control.

#### 2.6.4. Alamar Blue™ Cell Viability Assay

The cell viability was tested against cancer cells incubated for 3 h at 37 °C with a 10% Alamar Blue™ solution (Thermo Fisher Scientific). U-251MG, A431, HepG2, Caco-2, and HEK293 cells were seeded at a density of 1 × 10^4^ cells/well (100 μL culture medium/well) into flat-bottom 96-well plates (Sarstedt AG & Co. KG, Germany) and at a density of 1 × 10^4^ cells/well (200 μL culture medium/well) into Nunclon™ Sphera™ 96-well low-attachment plates (Thermo Fisher Scientific) to conduct the 2D and 3D cell culture methods, as explained above.

In the 2D cell culture method, each cell line (U-251MG, A431, HepG2, Caco-2, and HEK293 cells) was treated with samples of the desired concentration and incubated for 48 h and 120 h in the abovementioned conditions. Based on the results of the 2D cell culture, the U-251MG cells were selected using 3D tumour spheres to examine the toxicity levels in the samples with the desired concentration.

Following the above procedure, the 2D cells were washed with sterile phosphate-buffered saline (PBS) and a dye consisting of of 10% Alamar Blue™ solution and 90% Dulbecco’s Modified Eagle’s Medium high-glucose solution was added; they were then incubated for 3 h at 37 °C. During the 3D cell culture, after post-treatment incubation, the tumour spheres were washed with PBS, trypsinised using 0.25% *w*/*v* trypsin–EDTA solution, and incubated for 3 h at 37 °C with a 10% Alamar Blue™ solution. Fluorescence was measured using an excitation wavelength of 530 nm and an emission wavelength of 590 nm with a Varioskan^TM^ LUX multimode microplate reader (Thermo Fisher Scientific, Massachusetts, USA). All experiments consisted of at least three independent tests with a minimum of 30 replicates per experiment, which were statistically analysed.

#### 2.6.5. Live/Dead Cell Analysis Using Propidium Iodide (PI) for Flow Cytometry

The same procedure as for the 2D cell culture method was followed for flow cytometry. Based on the IC_50_ values, the concentration of each sample was selected against U-251MG and A431 cells. After being treated with the samples, the plates were incubated at 37 °C with 5% CO_2_ for 6 days. The medium was then removed, and the cells were washed with PBS and trypsinised, following PI staining for flow cytometry. The cells were then collected from the samples into a single centrifuge tube for centrifugation at 250 g for 5 min. The supernatant was aspirated, and the pellet was reconstituted in PBS (0.5% *w*/*v*). PI was then added to each cell suspension at 10 μg/mL and they were incubated in dark conditions for 1 min. Fluorescence was measured using the Beckman Coulter CytoFLEX Flow Cytometer (Beckman Coulter, Inc., Brea, USA) with a blue laser at 488 nm.

### 2.7. Wound-Healing Assay for Cell Migration

The U-251MG cell line was selected for the scratch assay to evaluate the wound-healing effects of the samples, as described by Shanmugapriya et al. [8,9]. Cells were cultured in Dulbecco’s Modified Eagle’s medium (DMEM) high glucose supplemented with 10% foetal bovine serum (FBS) and 1% penicillin–streptomycin as antibiotics and maintained in a humidified incubator containing 5% CO_2_ at 37 °C in a standard flask. Cells were subcultured with 0.25% *w*/*v* trypsin–EDTA solution until they reached 80–90% confluence. The wound-healing assay was used to assess cell migration in the samples. The cells were subcultured at the desired density of 5 × 10^5^ cells/well in a 6-well plate and incubated overnight at 37 °C with 5% CO_2_. A scratch was created in the centre of the well and washed with PBS. The cells were then treated with different concentrations of the samples and serially diluted from 1% to 0.0078%, whereas 20% dimethyl sulfoxide (DMSO) was used as a positive control and media were used as a negative control; following this, the cells were further incubated. Images were captured at 0 h and 24 h incubation with an optical microscope. All experiments were performed in triplicate. The level of cell migration was calculated using ImageJ (IJ, 1.46r) and quantified using Equation (3).

(3) $$Wound\;closure\;rate\;(\%)=\frac{Migration\;cell\;surface\;area}{Total\;surface\;area}\times 100\;$$

### 2.8. Statistical Analysis

All experiments were performed in triplicate, independently of each other, with a minimum of three replicates (n = 9) per experiment. All studies using seaweed samples were evaluated using statistical analysis with GraphPad Prism version 10.2.0 for Windows, GraphPad Software LLC, Boston, MA, USA. The significance level was determined by *p*-values. Dose–response curves were obtained using nonlinear regression. Data are presented as percentages, and the error bars of all figures adopt the standard error of the mean (SEM). Multiple comparison analyses using two-way analysis of variance (ANOVA) with post hoc Tukey’s tests were performed. The CytExpert software 2.4 (Version 2.4.0.28) was used for the flow cytometry analysis, and the mean of FITC-A was used to plot the values in dot plots and histograms.

## 3. Results and Discussion

### 3.1. Antioxidant Activity

The antioxidant potential was analysed using two specific chemical methods, namely DPPH and FRAP. Both of these methods are widely used to determine/measure the ability of bioactive compounds to act as free radical scavengers or hydrogen donors and to evaluate the antioxidant activity of samples. In addition, they can also be used to quantify antioxidants in complex biological systems [9].

**Figure 1:**
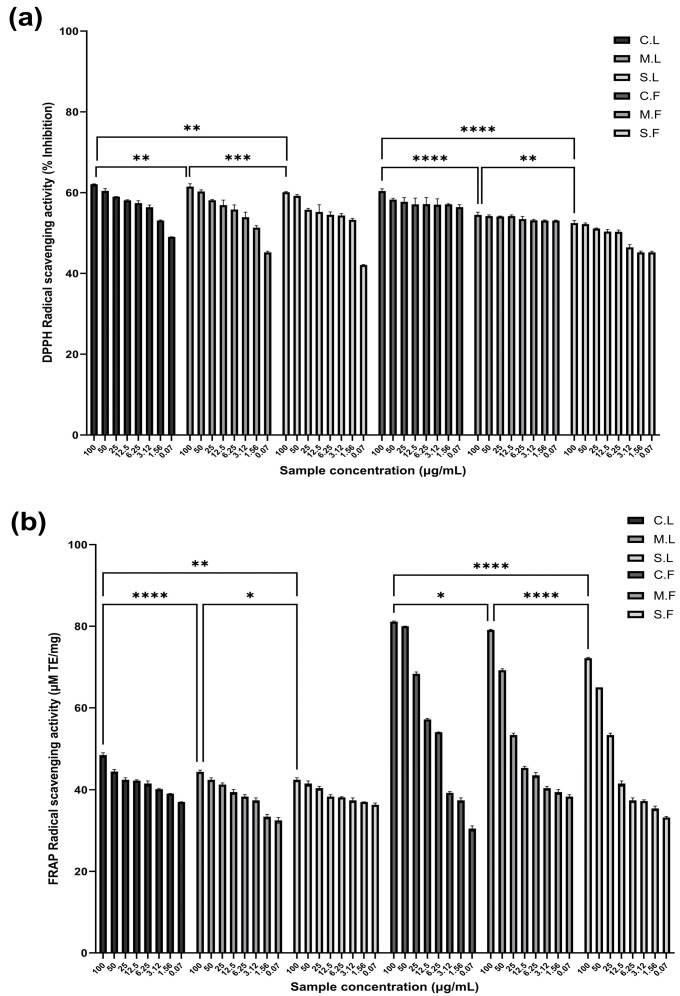
Bioactive properties of laminarin and fucoidan samples in different antioxidant assays. (**a**) 1,1-diphenyl-2-picryl-hydrazyl (DPPH) activity and (**b**) ferric-reducing antioxidant power (FRAP) assays at different concentrations. The graph shows the sample concentration in µg/mL on the X-axis and the percentage inhibition of antioxidant values on the Y-axis. All values represent the means of triplicate results (n = 3, Mean ± S.D.). Significant statistical differences in the antioxidant potential are represented as * *p* < 0.05, ** *p* < 0.01, *** *p* < 0.001, **** *p* < 0.0001. The seaweed samples used for the study were crude laminarin (C.L.), an MWCO 10 kDa laminarin fraction (M.L.), a Sigma laminarin standard (S.L.), crude fucoidan (C.F.), an MWCO 10 kDa fucoidan fraction (M.F.), and a Sigma fucoidan standard (S.F).

The DPPH radical scavenging activity of the crude and molecular weight cut-off samples of laminarin and fucoidan was observed to increase in a concentration-dependent manner compared with the controls (Figure 1a). The samples were tested at different concentrations using sterile distilled water as a solvent and serially diluted from 1 to 0.078 µg/mL. The results were analysed, and the values are expressed in triplicate, with the mean and SD, respectively. The DPPH scavenging values were determined based on the percentage inhibition using the calculation formula and were found to be 62.08%, 61.49%, and 60.1% for C.L., M.L., and S.L. and 60.39%, 54.49%, and 52.45% for C.F., M.F., and S.F., respectively. The FRAP values for each sample were found to be 48.41 µM TE/mg, 44.30 µM TE/mg, and 42.35 µM TE/mg for C.L., M.L., and S.L. and 81.16 µM TE/mg, 79.11 µM TE/mg, and 72.16 µM TE/mg for C.F., M.F., and S.F., respectively.

DPPH^•^ radicals may be neutralised either by direct reduction via electron transfer or by radical quenching via hydrogen atom transfer [32]. The FRAP assay is based on electron transfer, serving to evaluate the antioxidant potential. The FRAP values measure the ability to reduce the TPTZ–Fe (III) complex to the TPTZ–Fe (II) complex [14]. The FRAP values of the seaweed samples are shown in Figure 1b. The results showed higher FRAP values (µM TE/mg) for the crude laminarin and molecular weight cut-off fucoidan samples than other samples compared to the controls. Additionally, all samples had higher reducing potential than the reference antioxidants used in this study. Regarding the reducing ability measured with the FRAP assay, the samples ranged from higher to lower concentrations. Specifically, this difference depends on their composition, which influences their bioactivity [17,22]. The results showed a strong association between the DPPH radical scavenging and FRAP antioxidant activity of the seaweed samples. Additionally, the high antioxidant activity observed in brown seaweed extracts could be due to the presence of both polyphenols and polysaccharide compounds. Similar results were observed in many recent studies [20,32,41,42], indicating the high antioxidant potential of sulphated polysaccharides from seaweed. A lower molecular weight and higher reducing sugar content enhance the biofunctional activity of seaweed bioactives [32,43]. Regarding brown seaweed polysaccharides, laminarin and fucoidan have been reported to have biological and pharmacological activity and be involved in specific human diseases, such as cancer, severe brain and skin infections, neurodegeneration, and metabolic disorders [29,44].

### 3.2. Anti-Inflammatory Activity

**Figure 2:**
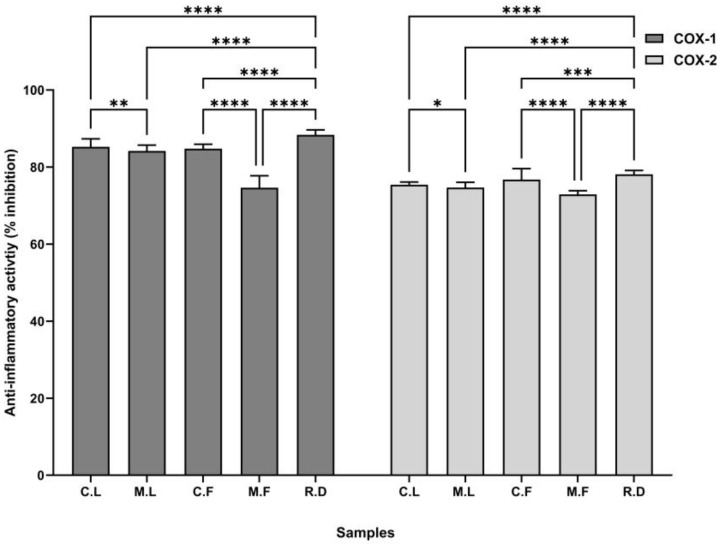
Effects of anti-inflammatory activity of seaweed samples with COX-1 and COX-2. All values represent the means of triplicate results and are expressed as the mean ± SD. Statistically significant differences in antioxidant potential are represented as * *p* < 0.05, ** *p* < 0.01, *** *p* < 0.001, **** *p* < 0.0001. Seaweed samples used were crude laminarin (C.L.), MWCO 10 kDa laminarin fraction (M.L.), crude fucoidan (C.F.), MWCO 10 kDa fucoidan fraction (M.F.), and reference drug (diclofenac sodium) as R.D.

The anti-inflammatory activity of the seaweed samples was analysed using the COX-1 and COX-2 enzymes. The anti-inflammatory activity of crude laminarin (C.L.), the MWCO 10 kDa laminarin fraction (M.L.), crude fucoidan (C.F.), the MWCO 10 kDa fucoidan fraction (M.F.), and the reference drug (diclofenac sodium) with COX-1 and COX-2 is shown in Figure 2. The results were 85.25% and 75.44% higher for crude laminarin and 84.77% and 76.75% higher for crude fucoidan than the molecular weight cut-off samples, as compared with the reference standard (Figure 2). The IC_50_ and selectivity index (SI) values of the samples with COX-1 and COX-2 are shown in Table 1. The anti-inflammatory effects of seaweed samples are associated with the reduction of nitric oxide, free radicals, and pro-inflammatory cytokines. These anti-inflammatory results highlight the therapeutic potential of laminarin and fucoidan.

There is increased interest in brown seaweed as a pharmacological source with bioactive constituents due to its anticancer and antioxidant activity. Seaweed polysaccharides have been reported to have various pharmacological properties, such as antioxidant, antimicrobial, anti-inflammatory, and anticoagulant activity [19,37,45]. Green technologies such as conventional extraction and ultrasound extraction, used in the extraction of crude samples, may enhance the inhibitory effects in the COX inhibitor screening assay compared with commercial standards. Many research studies have reported the anti-inflammatory effects of Laminaria species [36,37]. The anti-inflammatory mechanisms include the production of prostaglandins and the inhibition of inflammatory reactions in enzyme synthesis [23]. Seaweed samples can also relieve symptoms related to human gastrointestinal disorders and exhibit other anti-inflammatory health benefits by inhibiting COX enzymes [25,37].

### 3.3. Antidiabetic Activity

**Figure 3:**
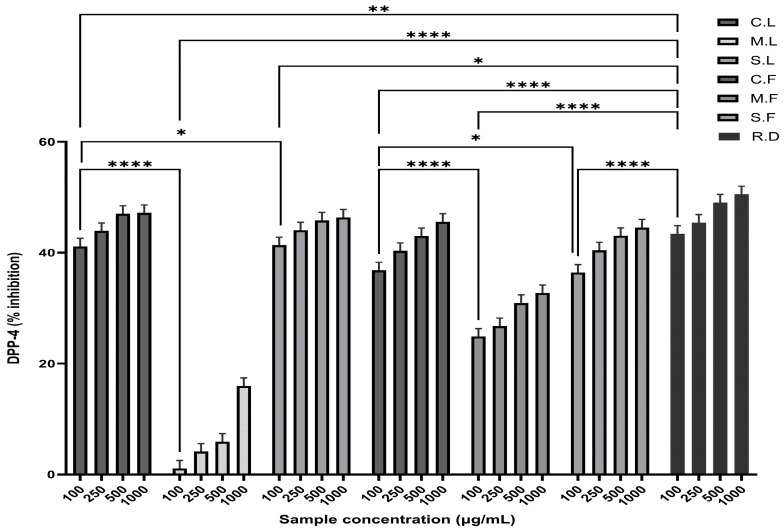
Screening of seaweed extracts for antidiabetic activity using dipeptidyl peptidase-4 (DPP-4) inhibitory activity. The seaweed samples were analysed for the inhibition of DPP-4 at different concentrations. All data represent the means of triplicate results and are represented as the mean ± SD. Statistically significant differences are represented with *p*-values (* *p* < 0.05, ** *p* < 0.01, **** *p* < 0.0001; n = 9). Seaweed samples used for the study were crude laminarin (C.L.), MWCO 10 kDa laminarin fraction (M.L.), Sigma laminarin standard (S.L.), crude fucoidan (C.F.), MWCO 10 kDa fucoidan fraction (M.F.), Sigma fucoidan standard (S.F.), and reference drug (R.D.).

The antidiabetic activity of the seaweed samples was analysed using the DPP-4 inhibition assay at different concentrations. Greater inhibition was found for the C.L. and C.F. samples than the M.L. and M.F. samples. The highest inhibition values observed were 46.84% for C.L., 15.29% for M.L., 45.70% for S.L., 44.91% for C.F., 34.07% for M.F., and 43.87% for S.F. compared to the reference standard, sitagliptin, with a value of 49.87% (Figure 3). As a result, the most promising crude seaweed extracts showed high DPP-4 inhibitory activity in a concentration-dependent manner.

DPP-4 inhibitors have a role in diabetic therapeutic treatment. Alpha-glucosidase and alpha-amylase, together with lipids, are involved in the digestion of carbohydrates, in addition to the activity of DPP-4 enzymes in the gastrointestinal tract. This mechanism of action results in the breakdown of carbohydrates into glucose molecules [26,29]. Suppressors delay carbohydrate digestion, which causes a reduction in the glucose absorption rate and reduces the rise in the plasma glucose level [30,31]. Much research has focused on the effective and safe inhibition of DPP-4 enzymes for the development of drugs for the management of diabetes. The in vitro evaluation of the seaweed samples demonstrated that the crude samples exhibited high DPP-4 inhibitory potential and hence potential for significant antidiabetic effects.

### 3.4. Cytotoxicity of Laminarin and Fucoidan in Cancer Cells in 2D and 3D Cell Culture Models

**Figure 4:**
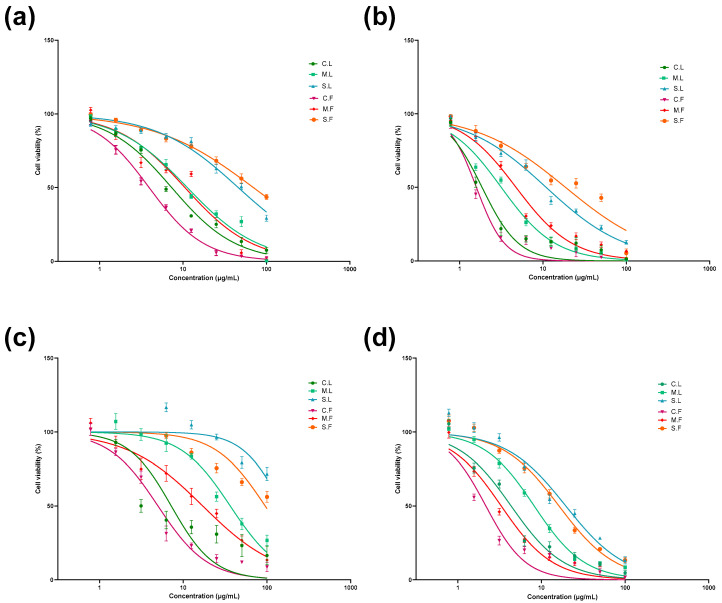
Cytotoxicity effects of different seaweed extracts in U-251MG human glioblastoma multiforme cells (**a**,**b**) and A431 human epidermoid carcinoma cells (**c**,**d**) at 2 days and 6 days post-treatment incubation. All values are expressed as the mean ± SD. Significant differences were analysed. Seaweed samples used for study were crude laminarin (C.L.), MWCO 10 kDa laminarin fraction (M.L.), Sigma laminarin standard (S.L.), crude fucoidan (C.F.), MWCO 10 kDa fucoidan fraction (M.F.), and Sigma fucoidan standard (S.F.).

**Figure 5:**
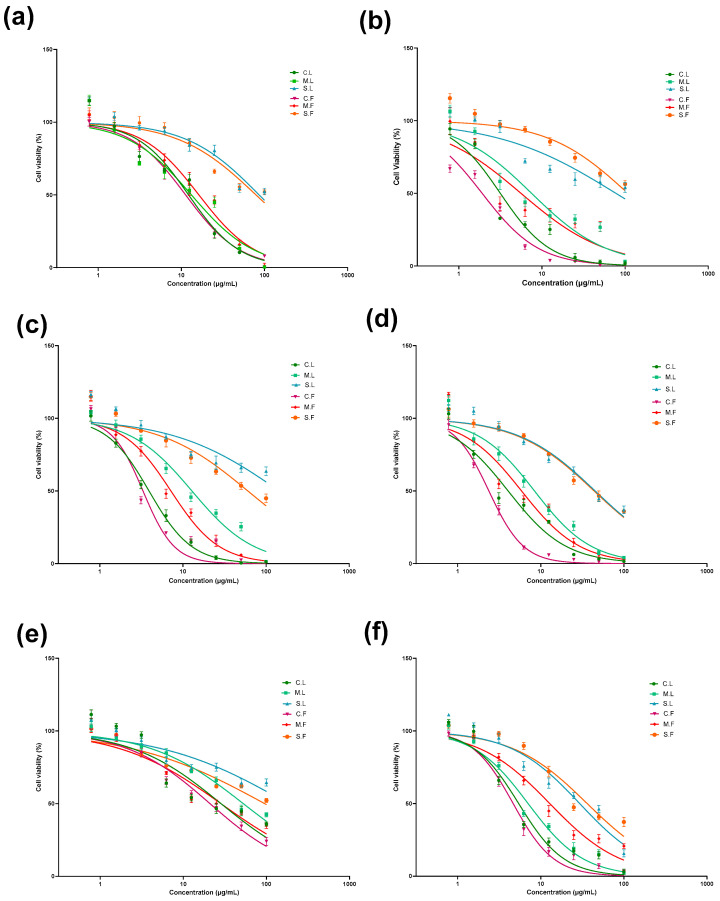
Cytotoxicity effects of different seaweed extracts in HepG2 human hepatoma cells (**a**,**b**), Caco-2 human colorectal adenocarcinoma cells (**c**,**d**), and HEK293 human embryonic kidney cells (**e**,**f**) at 2 days and 6 days of post-treatment incubation. All values are expressed as the mean ± SD. Significant differences were analysed. Seaweed samples used for study were crude laminarin (C.L.), MWCO 10 kDa laminarin fraction (M.L.), Sigma laminarin standard (S.L.), crude fucoidan (C.F.), MWCO 10 kDa fucoidan fraction (M.F.), and Sigma fucoidan standard (S.F.).

**Figure 6:**
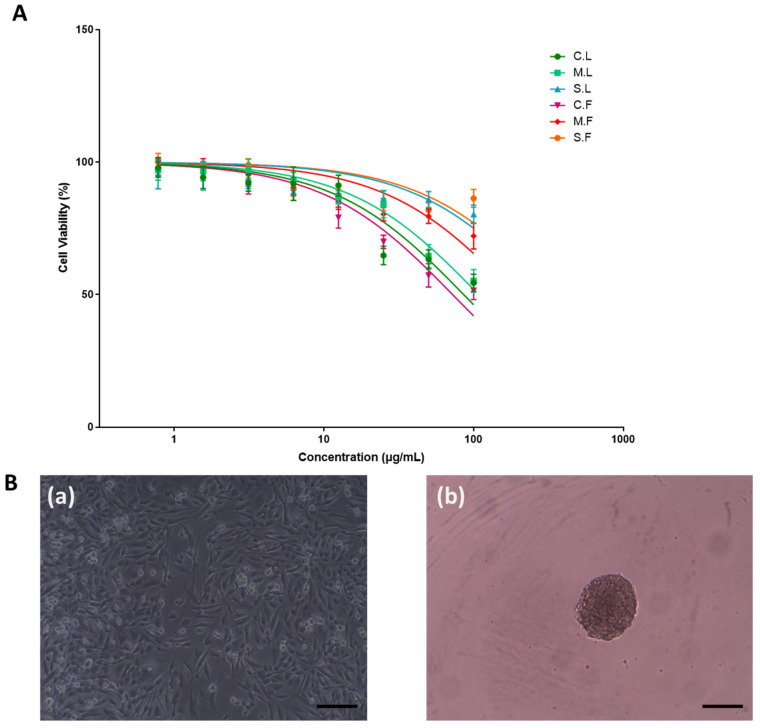
(**A**) Effects of U-251MG human glioblastoma 3D tumour spheres in in vitro cell culture model. (**B**) A graph representing different seaweed extracts at 6 days post-treatment incubation. (**a**) Image of U-251MG 2D cells and (**b**) 3D tumour spheres in low adhesion using an optical microscope with scale bar of 100 μm. All values are expressed as the mean ± SD. Significant differences were analysed. Seaweed samples used for study were crude laminarin (C.L.), MWCO 10 kDa laminarin fraction (M.L.), standard laminarin (S.L.), crude fucoidan (C.F.), MWCO 10 kDa fucoidan fraction (M.F.), and standard fucoidan (S.F.).

The cytotoxicity of the seaweed extracts was examined using the Alamar Blue™ cell viability assay in order to evaluate the anticancer properties of laminarin and fucoidan [29]. The samples were treated against all cell lines (2D) with Alamar Blue dye for 2 days and 6 days and analysed for their cytotoxicity (Figure 4 and Figure 5), as well as their IC_50_ values and ranges (Table 2). Laminarin and fucoidan samples were treated using U-251MG 3D tumour spheroids. The 3D cytotoxicity effect was analysed 6 days after post-treatment incubation using the Alamar Blue cell viability assay (Figure 6), and their IC_50_ values are shown in Table 3. The two-way ANOVA with the post hoc Tukey’s test showed significant differences in cell viability between each concentration, post-treatment incubation period, and cell line used.

As obtained using the 2D cell culture method, the cytotoxicity of the different seaweed extracts against different cell lines at 2 to 6 days of post-treatment incubation is shown in Figure 4 and Figure 5. The IC_50_ values for all seaweed samples are shown in Table 2.

Three-dimensional cell culture models/systems have a strong ability to mimic tissue structures, either from single cells or co-cultures. In 3D models, cell–cell communication and differentiated cellular function are more physiologically relevant than in two-dimensional cell culture models [39]. Morphological features that cannot be affected by 2D cultures and that influence the behaviour of cancer cells and their microenvironments can be examined [46,47,48].

**Figure 7:**
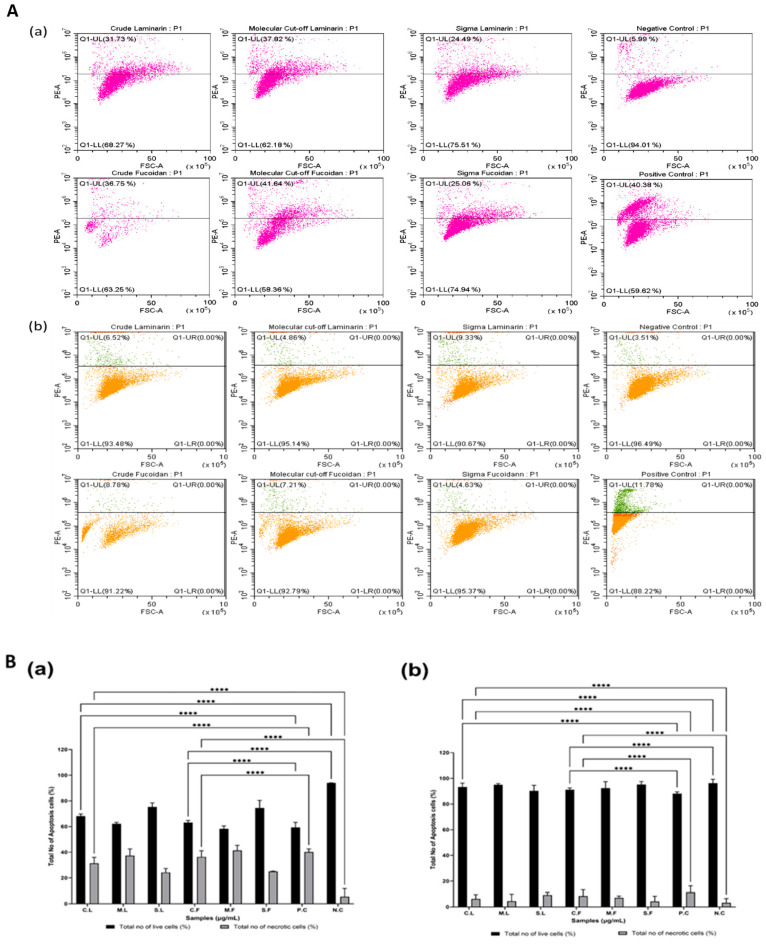
(**A**) Analysis of flow cytometry data for seaweed samples. (**a**) U-251MG human glioblastoma multiforme cells and (**b**) A431 human epidermoid carcinoma cells at 6 days post-treatment incubation. (**B**) Flow cytometry data of seaweed samples at 6 days post-treatment incubation. (**a**) A graph representing different samples in U-251MG human glioblastoma multiforme cells and (**b**) a graph representing different samples in A431 human epidermoid carcinoma cells. The graph shows the total number of apoptotic cells (%), represented as a bar chart, and we analysed the significant differences (**** *p* < 0.0001 vs. P.C. and N.C.). Seaweed samples used for study were crude laminarin (C.L.), MWCO 10 kDa laminarin fraction (M.L.), Sigma laminarin standard (S.L.), crude fucoidan (C.F.), MWCO 10 kDa fucoidan fraction (M.F.), and Sigma fucoidan standard (S.F.); positive and negative controls denoted as P.C. and N.C., respectively.

The cytotoxicity of the different seaweed extracts in U-251MG 3D tumour spheres at 6 days of post-treatment incubation are shown in Figure 6. The IC_50_ values for all seaweed samples are shown in Table 3. The two-way analysis of variance (ANOVA) with Tukey’s multiple comparisons test demonstrated that there was a significant difference (**** *p* < 0.001 vs. control) in tumour spheroid formation across the different seaweed extracts. The live and dead cells were confirmed using flow cytometry (Figure 7). The results confirmed that the crude seaweed samples showed higher programmed cell death than the molecular weight cut-off samples.

Different mechanisms of cancer drug resistance are involved in the development of targeted therapies. The tumour environment involves normal stromal cells (SC), the extracellular matrix (ECM), cytokines, and growth factors [5,8]. The ECM plays a key role in tumour progression, metastasis, angiogenesis, cytotoxicity resistance, and immune cell modulation. Cancer cells show uncontrolled proliferation and can suppress cell death through ROS, which act as secondary messengers. Drug resistance development is a serious problem in the field of cancer. Many researchers have focused their research on the effects of seaweed on cancer cells by using 2D monolayer cell cultures and confirmed their results using animal models. In the present study, the effects of laminarin and fucoidan on cancer cells were investigated using 2D monolayer cell cultures [49,50,51,52] and a 3D tumour sphere model. This is the first study to investigate laminarin and fucoidan extracts for the induction of cytotoxicity in 3D tumour spheroids and compare them with molecular weight cut-off samples. Tumour–tumour cell communication, tumour–stromal cell communication, and the tumour–ECM interface contributes to direct cell interactions mediated by cancer drug resistance [40,53]. A U-251MG 3D cell culture model can enable cell–cell and cell–ECM interactions in all three dimensions and mimic the diffusion-limited distribution of oxygen, nutrients, metabolites, and signalling molecules common in the in vivo microenvironments of tumours [54,55]. The present study demonstrated that the cancer cells used were resistant to the cellular stresses and agents/drugs/samples provided through cell mechanisms in cancer drug resistance.

Specifically, the seaweed extracts tested against different cell lines demonstrated a dose-dependent pattern in the cell proliferation rate [8,48]. The highest cytotoxicity observed in the seaweed samples may have been due to the increased reactive oxygen and nitrogen species, which play a major role in cancer cytotoxicity [5]. The results also proved that the extracted laminarin could induce toxicity in cancer cells and minimise the toxic effects on normal cells. There are several mechanisms involved, including the inactivation of the drug, multidrug resistance, the inhibition of cell death (apoptosis suppression), changes in drug metabolism, epigenetic and drug targets, enhanced DNA repair, and gene amplification, which cause resistance to the specific treatment methods involved. Based on the present results, these mechanisms need to be studied in further research.

In sulphate-modified laminarin extracts at 1.6 mg/mL, a decrease in cell proliferation (by 86%) was observed by Ji et al. [56]. Laminarin has strong potential to suppress cell viability, increase cell cycle arrest, and induce apoptosis in a dose-dependent manner [48]. Many studies have reported that laminarin inhibits cell growth, cell death by DNA fragmentation, reactive oxygen species production, and cell cycle progression in ovarian cancer cells [47]. Laminarin has a significant effect on dendritic cell maturation and anticancer immunity activation and was investigated as a novel immune stimulatory molecule for cancer immunotherapy [57]. The assay used was performed to assess the cytotoxicity of the sample, which plays a pivotal role in the immune system and tumour microenvironment. Specifically, the bioactive compounds in the samples may trigger cytotoxic effects, either through the activation of an apoptotic route or through a cytostatic effect that stops the cellular cycle.

### 3.5. Wound-Healing Assay for Cell Migration

**Figure 8:**
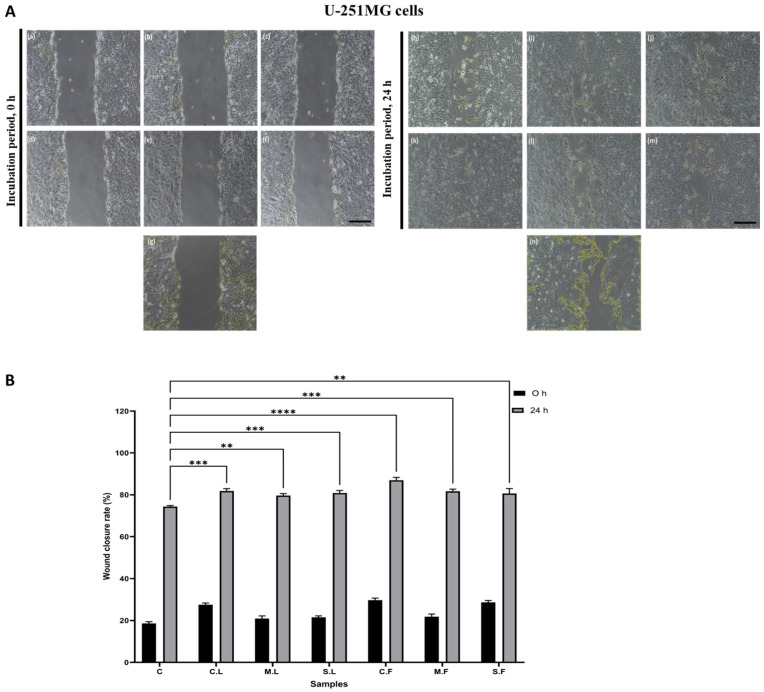
(**A**) Microscopic images captured at 0 h (a-g) and 24 h (h-n) after cell wounding in U-251MG cells and (**B**) wound closure rate (%) (n = 3; ** *p* < 0.01, *** *p* < 0.001, **** *p* < 0.0001 vs. control at 24 h). Seaweed samples used for study were crude laminarin (C.L., (**a**,**h**)), MWCO 10 kDa laminarin fraction (M.L., (**b**,**i**)), Sigma laminarin standard (S.L., (**c**,**j**)), crude fucoidan (C.F., (**d**,**k**)), MWCO 10 kDa fucoidan fraction (M.F., (**e**,**l**)), and Sigma fucoidan standard (S.F., (**f**,**m**)), compared with control (**g**,**n**).

A wound-healing assay for cell migration was performed to analyse the seaweed samples with U-251MG cells (Figure 8). A wound scratch area was captured at 0 h and 24 h in treated and controlled samples. The percentages of wound closure observed were 82.6 ± 0.3% for C.L. and 87.9 ± 2.6% for C.F. The results confirmed that the crude samples inhibited cell migration to a larger extent in U-251MG cells than the molecular cut-off samples due to their potential effects on cell viability and toxicity against cancer cells.

Recent studies have investigated the extraction and engineering of biodegradable biomaterials from marine sources for the development of natural skin products in cosmetics applications, including skin tissue regeneration [29,58]. Laminarin and fucoidan have many advantages for biomedical applications, including low cellular toxicity, biodegradability, and biocompatibility, with a diverse array of bioactivity that could be potentially employed in the wound-healing process [9,59,60,61]. The immunostimulatory activity influencing wound healing is mediated by cell surface receptors via the direct and direct activation of cytokines, keratinocytes, and fibroblasts [9].

Previous studies report that laminarin enhances wound repair and stimulates tissue regeneration, collagen deposition, and re-epithelialisation [60,61]. The bioactivity and physicochemical characteristics of fucoidan have been demonstrated to include antitumour, immunomodulatory, and antiviral effects. Fucoidan may also assist in the development of effective treatments for tissue engineering and other skincare applications [59,60]. In previous studies, seaweed polysaccharides with different molecular weights were compared with crude samples and shown to be potent inducers of wound closure and beneficial in treating burns by improving cell migration and proliferation during wound repair [59,60].

## 4. Conclusions and Future Prospects

All laminarin and fucoidan samples investigated were demonstrated to have antioxidant, anti-inflammatory, and antidiabetic activity and could contribute to cancer cell death and inhibit cell proliferation. These results were demonstrated using standard assays in the present study. The crude laminarin and fucoidan samples showed the strongest cytotoxic effects against different cancer cells using 2D and 3D cell culture models of U-251MG glioblastoma tumour spheres. This study suggests that seaweed bioactives offer alternatives in treating different types of cancer and in biomedical applications.

Further research is necessary to demonstrate the biological potential of laminarin and fucoidan bioactives for glioblastoma, cancer, and other related diseases. Additionally, further studies are required to investigate the molecular reduction of seaweed bioactives and their use as immunomodulatory and therapeutic agents for drug delivery and in food and biomedical applications.

## Figures and Tables

**Table 1:** Screening of anti-inflammatory activity of seaweed samples against COX-1 and COX-2.

Sample	IC_50_ Value (µg/mL)		Selectivity Index, SI
	COX-1	COX-2	
C.L.	48.96	42.74	1.06
M.L.	46.35	40.06	0.88
C.F.	47.41	42.27	1.58
M.F.	44.59	40.01	0.83
R.D.	49.64	45.19	1.07

Seaweed samples used were crude laminarin (C.L.), MWCO 10 kDa laminarin fraction (M.L.), crude fucoidan (C.F.), MWCO 10 kDa fucoidan fraction (M.F.), and reference drug (diclofenac sodium) as R.D.

**Table 2:** The 2D cell culture results: summary of IC_50_ values and IC_50_ ranges in seaweed extracts obtained with 2 days and 6 days of treatment in U-251MG, A431, HepG2, Caco-2, and HEK293.

Sample	Cell Line	2 Days		6 Days	
		IC_50_ (µg/mL)	IC_50_ Range	IC_50_ (µg/mL)	IC_50_ Range
C.L.	U-251MG	0.729	0.689 to 0.772	0.185	0.170 to 0.202
	A431	0.717	0.242 to 2.351	0.433	0.392 to 0.479
	HepG2	1.219	1.097 to 1.354	0.315	0.283 to 0.351
	Caco-2	0.390	0.373 to 0.408	0.404	0.365 to 0.448
	HEK293	2.841	2.323 to 3.530	0.542	0.490 to 0.601
M.L.	U-251MG	1.124	1.040 to 1.214	0.185	0.296 to 0.334
	A431	3.635	2.333 to 6.050	0.818	0.781 to 0.857
	HepG2	1.318	1.152 to 1.508	0.749	0.638 to 0.882
	Caco-2	1.283	1.186 to 1.389	0.840	0.767 to 0.920
	HEK293	5.267	4.833 to 5.770	0.703	0.649 to 0.763
S.L.	U-251MG	4.591	4.287 to 4.933	0.185	1.061 to 1.159
	A431	15.26	6.865 to 15.39	1.955	1.772 to 2.159
	HepG2	8.619	7.396 to 10.32	7.755	6.062 to 10.45
	Caco-2	14.47	10.67 to 21.64	4.515	4.094 to 5.006
	HEK293	18.29	13.57 to 26.81	2.936	2.611 to 3.310
C.F.	U-251MG	0.391	0.377 to 0.406	0.185	0.149 to 0.168
	A431	0.498	0.458 to 0.542	0.216	0.192 to 0.244
	HepG2	1.123	1.073 to 1.176	0.190	0.177 to 0.204
	Caco-2	0.330	0.303 to 0.362	0.237	0.230 to 0.244
	HEK293	2.092	1.875 to 2.342	0.465	0.438 to 0.494
M.F.	U-251MG	1.052	0.933 to 1.184	0.185	0.440 to 0.511
	A431	1.707	1.548 to 1.883	0.329	0.304 to 0.357
	HepG2	1.622	1.492 to 1.763	0.559	0.461 to 0.679
	Caco-2	0.717	0.654 to 0.786	0.586	0.512 to 0.672
	HEK293	2.835	2.500 to 3.235	1.282	1.167 to 1.412
S.F.	U-251MG	6.972	6.585 to 7.404	0.185	1.621 to 2.145
	A431	9.520	4.088 to 11.37	1.601	1.517 to 1.731
	HepG2	7.896	6.924 to 9.164	10.80	9.095 to 13.30
	Caco-2	6.046	5.166 to 7.211	4.376	4.035 to 4.763
	HEK293	9.544	7.982 to 11.73	3.614	3.238 to 4.056

Seaweed samples used for study were crude laminarin (C.L.), MWCO 10 kDa laminarin fraction (M.L.), standard laminarin (S.L.), crude fucoidan (C.F.), MWCO 10 kDa fucoidan fraction (M.F.), and standard fucoidan (S.F.).

**Table 3:** The 3D cell culture results: summary of IC_50_ and IC_50_ ranges in seaweed extracts obtained at 6 days of treatment in U-251MG cells.

Sample	6 Days	
	IC_50_ Value (µg/mL)	IC_50_ Range
C.L.	8.583	7.639 to 9.644
M.L.	10.82	9.991 to 11.71
S.L.	30.00	24.60 to 36.60
C.F.	7.237	6.625 to 7.906
M.F.	18.98	16.58 to 21.73
S.F.	33.51	27.01 to 41.56

Seaweed samples used for study were crude laminarin (C.L.), MWCO 10 kDa laminarin fraction (M.L.), Sigma laminarin standard (S.L.), crude fucoidan (C.F.), MWCO 10 kDa fucoidan fraction (M.F.), and Sigma fucoidan standard (S.F.).

## Data Availability

The original contributions presented in the study are included in the article, further inquiries can be directed to the corresponding author.

## References

[1] World Health Organization (2020). WHO Report on Cancer: Setting Priorities, Investing Wisely and Providing Care for All.

[2] Sung H., Ferlay J., Siegel R.L., Laversanne M., Soerjomataram I., Jemal A., Bray F. (2021). Global cancer statistics 2020: GLOBOCAN estimates of incidence and mortality worldwide for 36 cancers in 185 countries. CA A Cancer J. Clin..

[3] Gultekin M., Ramirez P.T., Broutet N., Hutubessy R. (2020). World Health Organization call for action to eliminate cervical cancer globally. Int. J. Gynecol. Cancer.

[4] Ferlay J., Colombet M., Soerjomataram I., Parkin D.M., Piñeros M., Znaor A., Bray F. (2021). Cancer statistics for the year 2020: An overview. Int. J. Cancer.

[5] Wanigasekara J., Barcia C., Cullen P.J., Tiwari B., Curtin J.F. (2022). Plasma induced reactive oxygen species-dependent cytotoxicity in glioblastoma 3D tumourspheres. Plasma Process. Polym..

[6] Perillo B., Di Donato M., Pezone A., Di Zazzo E., Giovannelli P., Galasso G., Castoria G., Migliaccio A. (2020). ROS in cancer therapy: The bright side of the moon. Exp. Mol. Med..

[7] Cotas J., Pacheco D., Gonçalves A.M., Silva P., Carvalho L.G., Pereira L. (2021). Seaweeds’ nutraceutical and biomedical potential in cancer therapy: A concise review. J. Cancer Metastasis Treat..

[8] Shanmugapriya K., Kim H., Kang H.W. (2020). Epidermal growth factor receptor conjugated fucoidan/alginates loaded hydrogel for activating EGFR/AKT signaling pathways in colon cancer cells during targeted photodynamic therapy. Int. J. Biol. Macromol..

[9] Shanmugapriya K., Kim H., Kang H.W. (2020). Fucoidan-loaded hydrogels facilitates wound healing using photodynamic therapy by in vitro and in vivo evaluation. Carbohydr. Polym..

[10] Ouyang Y., Qiu Y., Liu Y., Zhu R., Chen Y., El-Seedi H.R., Chen X., Zhao C. (2021). Cancer-fighting potentials of algal polysaccharides as nutraceuticals. Food Res. Int..

[11] Luo M., Zhou L., Huang Z., Li B., Nice E.C., Xu J., Huang C. (2022). Antioxidant therapy in cancer: Rationale and progress. Antioxidants.

[12] Zhu X., Healy L., Wanigasekara J., Zhao M., Padamati R.B., Karuppusamy S., Curtin J.F., Sivagnanam S.P., Rai D.K., Sun D.-W. (2023). Characterisation of laminarin extracted from brown seaweed Laminaria digitata, using optimized ultrasound-and ultrafiltration-assisted extraction method. Algal Res..

[13] Zhong Q., Wei B., Wang S., Ke S., Chen J., Zhang H., Wang H. (2019). The antioxidant activity of polysaccharides derived from marine organisms: An overview. Mar. Drugs.

[14] Silva M.M.C.L., dos Santos Lisboa L., Paiva W.S., Batista L.A.N.C., Luchiari A.C., Rocha H.A.O., Camara R.B.G. (2022). Comparison of in vitro and in vivo antioxidant activities of commercial fucoidans from Macrocystis pyrifera, Undaria pinnatifida, and Fucus vesiculosus. Int. J. Biol. Macromol..

[15] Ummat V., Sivagnanam S.P., Rai D.K., O’Donnell C., Conway G.E., Heffernan S.M., Fitzpatrick S., Lyons H., Curtin J., Tiwari B.K. (2024). Conventional extraction of fucoidan from Irish brown seaweed Fucus vesiculosus followed by ultrasound-assisted depolymerization. Sci. Rep..

[16] Usov A.I., Bilan M.I., Ustyuzhanina N.E., Nifantiev N.E. (2022). Fucoidans of Brown Algae: Comparison of Sulfated Polysaccharides from Fucus vesiculosus and Ascophyllum nodosum. Mar. Drugs.

[17] Obluchinskaya E.D., Pozharitskaya O.N., Zakharov D.V., Flisyuk E.V., Terninko I.I., Generalova Y.E., Smekhova I.E., Shikov A.N. (2022). The Biochemical composition and antioxidant properties of Fucus vesiculosus from the Arctic region. Mar. Drugs.

[18] Ajjarapu S.M., Tiwari A., Kumar S. (2023). Applications and Utility of Three-Dimensional In Vitro Cell Culture for Therapeutics. Future Pharmacol..

[19] Amaro H.M., Pagels F., Tavares T.G., Costa I., Sousa-Pinto I., Guedes A.C. (2022). Antioxidant and Anti-Inflammatory Potential of Seaweed Extracts as Functional Ingredients. Hydrobiology.

[20] El-Beltagi H.S., Mohamed A.A., Mohamed H.I., Ramadan K.M., Barqawi A.A., Mansour A.T. (2022). Phytochemical and potential properties of seaweeds and their recent applications: A review. Mar. Drugs.

[21] Jayapala N., Perumal M.K., Baskaran R., Vallikannan B. (2022). Pharmacological Importance of Bioactive Molecules of Seaweeds. Sustainable Global Resources of Seaweeds Volume 2: Food, Pharmaceutical and Health Applications.

[22] Gabbia D., De Martin S. (2020). Brown seaweeds for the management of metabolic syndrome and associated diseases. Molecules.

[23] Liyanage N., Nagahawatta D., Jayawardena T.U., Jeon Y.-J. (2023). The Role of Seaweed Polysaccharides in Gastrointestinal Health: Protective Effect against Inflammatory Bowel Disease. Life.

[24] Rengasamy K.R., Mahomoodally M.F., Aumeeruddy M.Z., Zengin G., Xiao J., Kim D.H. (2020). Bioactive compounds in seaweeds: An overview of their biological properties and safety. Food Chem. Toxicol..

[25] Rocha D.H., Pinto D.C., Silva A. (2022). Macroalgae Specialized Metabolites: Evidence for Their Anti-Inflammatory Health Benefits. Mar. Drugs.

[26] Gunathilaka T.L., Samarakoon K., Ranasinghe P., Peiris L.D.C. (2020). Antidiabetic potential of marine brown algae—A mini review. J. Diabetes Res..

[27] Calderwood D., Rafferty E., Fitzgerald C., Stoilova V., Wylie A., Gilmore B.F., Castaneda F., Israel A., Maggs C.A., Green B.D. (2021). Profiling the activity of edible European macroalgae towards pharmacological targets for type 2 diabetes mellitus. Appl. Phycol..

[28] Unnikrishnan P., Suthindhiran K., Jayasri M. (2015). Antidiabetic potential of marine algae by inhibiting key metabolic enzymes. Front. Life Sci..

[29] Karuppusamy S., Rajauria G., Fitzpatrick S., Lyons H., McMahon H., Curtin J., Tiwari B.K., O’Donnell C. (2022). Biological Properties and Health-Promoting Functions of Laminarin: A Comprehensive Review of Preclinical and Clinical Studies. Mar. Drugs.

[30] Jayapala N., Toragall V., Kumar G., Chaudhari S.R., Baskaran V. (2022). Preparation, characterization, radical scavenging property and antidiabetic potential of laminarioligosaccharides derived from laminarin. Algal Res..

[31] Pozharitskaya O.N., Obluchinskaya E.D., Shikov A.N. (2020). Mechanisms of bioactivities of fucoidan from the brown seaweed Fucus vesiculosus L. of the Barents Sea. Mar. Drugs.

[32] Rajauria G., Ravindran R., Garcia-Vaquero M., Rai D.K., Sweeney T., O’Doherty J. (2021). Molecular characteristics and antioxidant activity of laminarin extracted from the seaweed species Laminaria hyperborea, using hydrothermal-assisted extraction and a multi-step purification procedure. Food Hydrocoll..

[33] Garcia-Vaquero M., Rajauria G., Tiwari B., Sweeney T., O’Doherty J. (2018). Extraction and yield optimisation of fucose, glucans and associated antioxidant activities from Laminaria digitata by applying response surface methodology to high intensity ultrasound-assisted extraction. Mar. Drugs.

[34] Dudonne S., Vitrac X., Coutiere P., Woillez M., Mérillon J.-M. (2009). Comparative study of antioxidant properties and total phenolic content of 30 plant extracts of industrial interest using DPPH, ABTS, FRAP, SOD, and ORAC assays. J. Agric. Food Chem..

[35] Henderson T., Nigam P.S., Owusu-Apenten R.K. (2015). A universally calibrated microplate ferric reducing antioxidant power (FRAP) assay for foods and applications to Manuka honey. Food Chem..

[36] Lu X., Dissanayake A.A., Xiao C., Gao J., Zhao M., Nair M.G. (2022). The edible seaweed *Laminaria japonica* contains cholesterol analogues that inhibit lipid peroxidation and cyclooxygenase enzymes. PLoS ONE.

[37] El-Ghoneimy A., Ahmed H. (2022). Anti-inflammatory activities of a sulfated polysaccharide isolated from the brown seaweed Padina boergesenii (Phaeophyceae, Dictyotaceae). SVU-Int. J. Vet. Sci..

[38] Khalid M.F., Rehman K., Irshad K., Chohan T.A., Akash M.S.H. (2022). Biochemical investigation of inhibitory activities of plant-derived bioactive compounds against carbohydrate and glucagon-like Peptide-1 metabolizing enzymes. Dose-Response.

[39] Wanigasekara J., Carroll L.J., Cullen P.J., Tiwari B., Curtin J.F. (2023). Three-Dimensional (3D) in vitro cell culture protocols to enhance glioblastoma research. PLoS ONE.

[40] Wanigasekara J., Cullen P.J., Bourke P., Tiwari B., Curtin J.F. (2022). Advances in 3D culture systems for therapeutic discovery and development in brain cancer. Drug Discov. Today.

[41] Bonfim-Mendonca P.d.S., Capoci I.R.G., Tobaldini-Valerio F.K., Negri M., Svidzinski T.I.E. (2017). Overview of β-glucans from laminaria spp.: Immunomodulation properties and applications on biologic models. Int. J. Mol. Sci..

[42] Michalak I., Tiwari R., Dhawan M., Alagawany M., Farag M.R., Sharun K., Emran T.B., Dhama K. (2022). Antioxidant effects of seaweeds and their active compounds on animal health and production–a review. Vet. Q..

[43] Choi J.-i., Kim H.-J., Lee J.-W. (2011). Structural feature and antioxidant activity of low molecular weight laminarin degraded by gamma irradiation. Food Chem..

[44] Zargarzadeh M., Amaral A.J., Custódio C.A., Mano J.F. (2020). Biomedical applications of laminarin. Carbohydr. Polym..

[45] Dhara S., Chakraborty K. (2022). Novel Furanyl-Substituted Isochromanyl Class of Anti-Inflammatory Turbinochromanone from Brown Seaweed Turbinaria conoides. Chem. Biodivers..

[46] Tian L., Li C.-M., Li Y.-F., Huang T.-M., Chao N.-X., Luo G.-R., Mo F.-R. (2020). Laminarin from seaweed (*Laminaria japonica*) inhibits hepatocellular carcinoma through upregulating senescence marker protein-30. Cancer Biother. Radiopharm..

[47] Bae H., Song G., Lee J.-Y., Hong T., Chang M.-J., Lim W. (2020). Laminarin-derived from brown algae suppresses the growth of ovarian cancer cells via mitochondrial dysfunction and ER stress. Mar. Drugs.

[48] Cabral E.M., Mondala J.R.M., Oliveira M., Przyborska J., Fitzpatrick S., Rai D.K., Sivagnanam S.P., Garcia-Vaquero M., O’Shea D., Devereux M. (2021). Influence of molecular weight fractionation on the antimicrobial and anticancer properties of a fucoidan rich-extract from the macroalgae Fucus vesiculosus. Int. J. Biol. Macromol..

[49] Santhanam R.C., Yacoob S.A.M., Venkatraman A. (2022). In vitro cytotoxicity assay of Fucoidan extracted from Turbinaria conoides against cancer cell lines MCF7, A549, and normal cell line L929. Braz. J. Pharm. Sci..

[50] Malhão F., Ramos A.A., Macedo A.C., Rocha E. (2021). Cytotoxicity of seaweed compounds, alone or combined to reference drugs, against breast cell lines cultured in 2D and 3D. Toxics.

[51] Langhans S.A. (2018). Three-dimensional in vitro cell culture models in drug discovery and drug repositioning. Front. Pharmacol..

[52] Vaamonde-García C., Capelo-Mera E., Flórez-Fernández N., Torres M.D., Rivas-Murias B., Mejide-Faílde R., Blanco F.J., Domínguez H. (2022). In Vitro Study of the Therapeutic Potential of Brown Crude Fucoidans in Osteoarthritis Treatment. Int. J. Mol. Sci..

[53] Lv D., Hu Z., Lu L., Lu H., Xu X. (2017). Three-dimensional cell culture: A powerful tool in tumor research and drug discovery. Oncol. Lett..

[54] Habanjar O., Diab-Assaf M., Caldefie-Chezet F., Delort L. (2021). 3D cell culture systems: Tumor application, advantages, and disadvantages. Int. J. Mol. Sci..

[55] Salinas-Vera Y.M., Valdés J., Pérez-Navarro Y., Mandujano-Lazaro G., Marchat L.A., Ramos-Payán R., Nuñez-Olvera S.I., Pérez-Plascencia C., López-Camarillo C. (2022). Three-dimensional 3D culture models in gynecological and breast cancer research. Front. Oncol..

[56] Ji C.-F., Ji Y.-B., Meng D.-Y. (2013). Sulfated modification and anti-tumor activity of laminarin. Exp. Ther. Med..

[57] Song K., Xu L., Zhang W., Cai Y., Jang B., Oh J., Jin J.-O. (2017). Laminarin promotes anti-cancer immunity by the maturation of dendritic cells. Oncotarget.

[58] George J., Thabitha A., Vignesh N., Manigandan V., Saravanan R., Daradkeh G., Qoronfleh M.W. (2021). Antiskin cancer and antioxidant activities of formulated agar from Brown seaweed Laminaria digitata (hudson) in dimethyl benzanthracene-induced Swiss albino mice. Int. J. Polym. Sci..

[59] Haggag Y.A., Abd Elrahman A.A., Ulber R., Zayed A. (2023). Fucoidan in pharmaceutical formulations: A comprehensive review for smart drug delivery systems. Mar. Drugs.

[60] Majtan J., Jesenak M. (2018). β-Glucans: Multi-functional modulator of wound healing. Molecules.

[61] Sellimi S., Maalej H., Rekik D.M., Benslima A., Ksouda G., Hamdi M., Sahnoun Z., Li S., Nasri M., Hajji M. (2018). Antioxidant, antibacterial and in vivo wound healing properties of laminaran purified from Cystoseira barbata seaweed. Int. J. Biol. Macromol..

